# Adaptation and study protocol of the evidence-based Make Better Choices (MBC2) multiple diet and activity change intervention for a rural Appalachian population

**DOI:** 10.1186/s12889-022-14475-0

**Published:** 2022-11-08

**Authors:** Nancy E. Schoenberg, Deanna Sherman, Angela Fidler Pfammatter, Michelle K. Roberts, Ming-Yuan Chih, Sarah C. Vos, Bonnie Spring

**Affiliations:** 1 Department of Behavioral Science, University of Kentucky, 760 Press Avenue, 468 Healthy Kentucky Research Building, Lexington, KY 40536 USA; 2 Department of Preventive Medicine, Northwestern University Feinberg School of Medicine, Chicago, IL USA; 3 Department of Anthropology, University of Kentucky, Lexington, KY USA; 4 Department of Health & Clinical Sciences, University of Kentucky, Lexington, KY USA; 5 Department of Health Management and Policy, University of Kentucky, Lexington, KY USA

**Keywords:** Rural populations, Technology, Exercise, Diet, Community-based participatory research, Mobile phone

## Abstract

**Background:**

Rural Appalachian residents experience among the highest prevalence of chronic disease, premature mortality, and decreased life expectancy in the nation. Addressing these growing inequities while avoiding duplicating existing programming necessitates the development of appropriate adaptations of evidence-based lifestyle interventions. Yet few published articles explicate how to accomplish such contextual and cultural adaptation.

**Methods:**

In this paper, we describe the process of adapting the Make Better Choices 2 (MBC2) mHealth diet and activity randomized trial and the revised protocol for intervention implementation in rural Appalachia. Deploying the NIH’s Cultural Framework on Health and Aaron’s Adaptation framework, the iterative adaptation process included convening focus groups (*N* = 4, 38 participants), conducting key informant interviews (*N* = 16), verifying findings with our Community Advisory Board (*N* = 9), and deploying usability surveys (*N* = 8), wireframing (*N* = 8), and pilot testing (*N* = 9. This intense process resulted in a comprehensive revision of recruitment, retention, assessment, and intervention components. For the main trial, 350 participants will be randomized to receive either the multicomponent MBC2 diet and activity intervention or an active control condition (stress and sleep management). The main outcome is a composite score of four behavioral outcomes: two outcomes related to diet (increased fruits and vegetables and decreased saturated fat intake) and two related to activity (increased moderate vigorous physical activity [MVPA] and decreased time spent on sedentary activities). Secondary outcomes include change in biomarkers, including blood pressure, lipids, A1C, waist circumference, and BMI.

**Discussion:**

Adaptation and implementation of evidence-based interventions is necessary to ensure efficacious contextually and culturally appropriate health services and programs, particularly for underserved and vulnerable populations. This article describes the development process of an adapted, community-embedded health intervention and the final protocol created to improve health behavior and, ultimately, advance health equity.

**Trial registration:**

ClinicalTrials.gov Identifier NCT04309461. The trial was registered on 6/3/2020.

**Supplementary Information:**

The online version contains supplementary material available at 10.1186/s12889-022-14475-0.

## Background

Appalachia, home to 26 million residents, extends across thirteen states from New York to Mississippi. Within this large region, the Central Appalachian area, including West Virginia, Eastern Kentucky, Southwest Virginia, East Tennessee and Western North Carolina is well known for its rurality, suboptimal socioeconomic and health status and sparse resources [1]. Compared with the US overall, for example, Appalachian Kentucky (KY) residents have almost twice the percentage of poverty (24.5% versus 13.4%), more than twice the premature death rate (12,028 versus 5,317 per 100,000), and substantially higher percentages of obesity (35.2% versus 27.4%, 6^th^ highest) and physical inactivity (32.8% versus 23.1%) [2–4]. Only 12% of Appalachian Kentuckians consume five or more servings of fruits and vegetables daily, compared with twice that percentage nationally [5]. Relatedly, Kentucky residents have elevated rates of cardiovascular disease (8^th^ highest mortality in US), diabetes (5^th^ highest mortality in the US), and the highest cancer rate in the nation [6–8]. This confluence of adverse circumstances and disease burden contributes to the region’s decreasing life expectancy, with a growing gap (0.6 year and 2.4 year difference in 1990–92 and 2009–13, respectively) between Appalachian residents and the US population overall. A recent report from the Appalachian Regional Commission indicated that years of potential life lost is 69% higher in Central Appalachia compared to the national average [2, 9].

Disease prevention through lifestyle modification has been well established [10], with diet and physical activity considered the most effective approaches to preventing and controlling chronic disease risk. Behavior change interventions that employ theoretically rigorous approaches offer the potential to improve health behavior and outcomes. One such intervention is Make Better Choices 2 (MBC2), a multicomponent mHealth program that involves use of (1) a smartphone application (app) that participants use to record what they eat and visually displays their daily diet and physical activity accomplishments relative to goals, (2) health coaching, (3) accelerometers (Fitbits), and (4) behavioral incentives [11]. Having diet and physical activity data transmitted regularly from the app and made visible on the coach’s dashboard holds participants accountable for monitoring and optimizing their behavior and enables the coach to tailor telephone coaching to the individual’s unique behavioral challenges. The program has produced large diet and physical activity improvements (*P* < 0.001), maintained over time [12]. Compared to a contact-matched control condition, participants in the MBC2 intervention improved diet and physical activity behaviors to achieve and maintain guideline recommended levels. At 9 months (6 months after the conclusion of weekly coaching), fruit and vegetable consumption had increased by 6.5 servings per day; saturated fat intake decreased by 3.6%; physical activity increased by 24.7 min per day; and sedentary behavior (leisure time, not including work or school screen time) decreased by 170.5 min per day [12].

Consistent with most other mHealth interventions, the initial MBC2 trial was conducted with urban dwellers. Such effective interventions have rarely, if ever, been implemented and evaluated among rural residents, although the limited available literature suggests mHealth as a feasible and acceptable intervention strategy among rural populations [13]. Evaluations of technology interventions are particularly absent within the Appalachian context, as researchers often assume that such interventions lack acceptability or feasibility. The few existing rural mHealth studies demonstrate critical shortcomings, including inadequately controlled research designs, limited inclusion of biomarkers, and non-significant or un-sustained behavioral improvements [14–16].

The great need for risk reduction in low-income rural settings, coupled with the scarcity of research on the effectiveness of evidence-based interventions in this population compels us to adapt, implement, and evaluate such interventions to achieve rural health equity. Adding to this imperative, the increasing use of mobile technology has created a viable channel to reach low-income rural residents in their home environment. Smartphones, sensors, and other technology with health-related functionality has expanded the capacity to perform remote behavioral monitoring and telehealth services [17, 18]. While broadband access in rural regions continues to be a barrier, (e.g., 21% and 10% of rural residents, respectively, report that internet connectivity constitutes a problem or a major problem) rural residents demonstrate extensive and increasing use of mobile technologies. For example, in a pre-pandemic poll (January 2019), 70% of rural respondents indicated that they use internet to obtain health information [19]. The Covid-19 pandemic not only highlighted the necessity of technology access but also increased and accelerated the uptake of mHealth technologies in rural communities. By early 2021, most residents of rural areas reported having a smart phone (80%), internet access (90%), and broadband internet connectivity (72%), representing a 5–10% increase since 2019 [20].

In this article, we describe our efforts to leverage this trend of increasing technology use by adapting an mHealth healthy lifestyle promotion intervention for rural Appalachians. The adapted mhealth randomized control behavioral trial involves a hybrid type 1 effectiveness design. We ultimately aim to assess the effectiveness of the adapted MBC2 for a rural Appalachian population, simultaneously examining implementation outcomes and cultural and contextual factors to establish the evidence base for adapted mHealth interventions in traditionally underserved rural populations.

## Methods

### Adaptation process and outcomes

#### Overview

This study takes place in Appalachian Kentucky, an area that includes 54 counties in the eastern part of the state. Data collection sites include community locations in Harlan County and Fayette County, Kentucky, USA. To ensure the fit of the evidence-based MBC2 intervention [12] for a rural population and setting, we employed an iterative cultural adaptation approach, engaging in several steps to assess the MBC2 intervention’s acceptability, feasibility, and need for cultural and contextual adaptation. Our overall model of adaptation was based on the NIH’s Cultural Framework of Health [21] and Aaron’s Adaptation framework [22]. With support from a $50,000 pilot grant through the University of Kentucky’s Center of Research in Obesity and Cardiovascular Disease, we conducted a series of focus groups (FG, *N* = 38) and key informant interviews (KII, *N* = 16). We verified our findings from the FG and KII and field-tested instruments with our Community Advisory Board (CAB, *N* = 9). We then engaged in wireframe testing (*N* = 8), followed by a usability survey (*N* = 8) with new participants. Revising the protocol and contents with each new activity, we then pilot tested the adapted MBC2 program with eligible participants (*N* = 9). We analyzed the results through template coding and descriptive statistics.

**Fig. 1:**
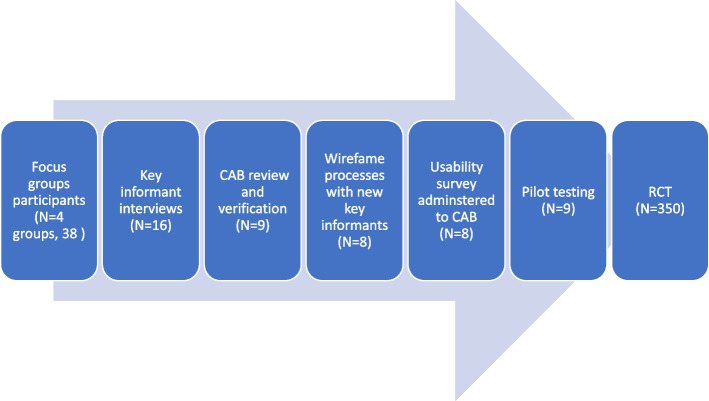
The Process of Adapting MBC2 to a Rural, Appalachian Population

We used this iterative process, shown in Fig. 1, to comprehensively assess and revise the processes of recruitment, retention, and assessment and the intervention components [23]. Figure 1 summarizes the adaptation process.

#### Focus group and key informant interviews

We used focus groups (FG) and key informant interviews (KII) to assess intervention feasibility and acceptability from the perspectives of rural Appalachian residents. Participants (FG, *N* = 38 in four focus groups; KII, *N* = 16) lived in six rural Appalachian counties, all of which are considered rural according to rural–urban commuting area (RUCA) Codes (7 [nonmetro, urban population of 2500–19,999, not adjacent to a metro area] to 9 [nonmetro, completely rural or less than 2500 population] [24]. Convenience sampling guided the selection of FG participants [25]. To ensure variability in education, income, and lifestyle behaviors while maintaining similarity to the overall Appalachian population, we employed maximum variation sampling, which aims to include a wide variety of participants despite having a modest sample size [26]. FG participants were recruited via community stakeholders through informal connections at work, church, and social service agencies. KII were conducted with individuals whose special expertise could provide particular insights (e.g., as a parent, an older adult, a tech sector worker, or a minimal user of technology). Purposive sampling was used to select KII from targeted organizations, such as churches and community centers. For both FG and KII, participant eligibility criteria included being age 18 years or older, an Appalachian resident, and willing and able to participate. Both FG and KII were conducted until thematic saturation was reached. Additional information about the FG and KII process is available elsewhere [27].

To assess the appropriateness and feasibility of the MBC2 program for Appalachian residents, we developed a slide show that displayed all program components. During the FGs, slides were presented about assessment schedules, general descriptions of the health coaches, frequency and dollar amount of incentives and, most especially, characteristics of the app. Participants observed and engaged with screen shots that displayed app features, including log in and home screen, profile setup, settings and information, and displays for charting main outcomes for the two intervention arms. After displaying each screen shot, we used a think aloud protocol [28] and audio-taped the participant’s responses. The interview guide for the KII was developed based on the Consolidated Framework for Implementation Research (CFIR) [29]. Specific questions were tailored to the participants’ expertise—for example, parents might be asked about optimal timing of telephone coaching related to competing childcare needs. Interviews were recorded and transcribed. Team members analyzed the transcripts according to pre-established codes, including: (1) overall usability, (2) whether or not (and why) participants would use the app, (3) problematic or confusing aspects, and (4) suggestions for improvement. Findings from FG and KII were merged to obtain a comprehensive perspective on adaptation of MBC2. These qualitative data were complemented by survey data that assessed satisfaction, likelihood of participation, accessibility, acceptability, and other feasibility constructs.

#### Findings

Participant feedback demonstrated a high level of acceptability: 90% reported that it was extremely or highly likely that MBC2 would be non-burdensome, potentially effective, ethical, and otherwise appropriate [30]. Feedback also provided evidence of feasibility: 92% indicated that MBC2 was either extremely likely or very likely to meet a community demand, work locally, and be delivered consistently with the original intervention when appropriately adapted [31].

As summarized in Table 1, participants recommended adaptations in several key areas to improve MBC2’s fit with the rural Appalachian context: eligibility, recruitment, and intervention programming. First, since rural communities, including Appalachian KY, have a disproportionately older population, participants suggested eliminating upper age restrictions. Additionally, because of the widespread practice and influence of multi-generational households in rural Appalachia, participants warned that eligibility criteria excluding more than one household member could discourage enrollment. Thus, we expanded inclusion criteria to include co-residing family members. Recruitment approaches also needed modification. Participants for the original MBC2 trial were recruited via posted advertising banners on mass transit (buses, trains), which does not exist in Appalachian Kentucky. Instead, word of mouth via close-knit community and support networks (including churches or Parent-Teacher Associations) were suggested as more promising ways to engage participants [32, 33]. Additionally, participants suggested using Facebook, as this and other social media approaches are widely used to share information in the Appalachian context irrespective of age. FG and KII participants also suggested developing recruitment materials that feature locally recognizable landmarks and local residents.

**Table 1:** Issues that required modification from original intervention

MBC2 component	Local challenge	Proposed adaptation
Eligibility criterion: age and household membership	Rural communities have older populations; multigenerational co-residence common.	Open up to all eligible adults 18+--no upper age limits; open to all co-residing adults.
Recruitment	Lack of urban recruitment resources. (e.g., public transit)	Use social media, community settings. (church, centers)
Intervention component: health coaches	Non-local coaches lack familiarity with available resources and local culture, undermining relevance and decreasing participant comfort. Insufficient and class divergent local health care providers.	Employ only lay, local coaches who know local norms, values, and community resources. Rigorously train local residents to promote capacity, expertise and sustainability.
Intervention component: coaching	Tight knit communities decrease enthusiasm for only individual-level activities.	Conduct quarterly group events.
Intervention component: App	Concern about data costs; less tech experience.	Special health coach training on data use/cost; conduct in-person user training session.
Intervention component: App messaging	Images lack resonance with local needs and population.	Reflect Appalachian context, with local images. Highlight success stories, fun activities.

Numerous modifications were suggested. Participants placed a premium on ensuring that coaches are local and lay people rather than health care professionals since local residents are more cognizant of local assets and limitations, including technology availability, data costs, and resources to support physical activity and healthy eating. Employing local lay residents was viewed as preferable to hiring health care providers and more sustainable. Not only are local health care providers few in number, but they also are often viewed as coming from more elite, less accessible backgrounds. Staffing with local residents was viewed as essential to ensure full awareness of local resources, social structure, and cultural beliefs (e.g., the community as extended family) [34]. Participants emphasized the need to ensure that coaches receive rigorous training to equip them with accurate knowledge and guide appropriate conduct.

Several additional programmatic adaptations were suggested. First, given the relative recency of technology use among middle aged and older Appalachians, participants recommended holding an in-person training on the app. During this training, participants suggested describing any and all potential expenses associated with using the app, since the local population tends to have low income and any hidden costs would undermine retention. Additionally, participants recommended group activities, including coming together as a community on a quarterly basis to encourage enrollment, retention, and programmatic success. (These recommendations were made prior to the start of the Covid pandemic). Sharing success stories featuring recognizable local settings and participants was viewed as a very promising approach. Concerns about privacy and confidentiality were minimal. We received input that Fitbits were the preferred physical activity monitor. Other components of the existing MBC2 program, including assessment periods, incentive payments, and instruments, were viewed as acceptable and not in need of modifications.

#### Verification with CAB

After incorporating these revisions into the programming, we convened our Community Advisory Board (CAB, *N* = 9), none of whom had participated in the FG or KII. To verify the appropriateness of the adaptations, we showed the CAB a revised slide deck that incorporated the above described protocol recommendations, employing the same think aloud process described above. The CAB voiced widespread agreement with the modifications suggested during the formative research, prompting progression to the next stage: wireframing.

#### Wireframing

Wireframing involves an app designer creating a simple two-dimensional schematic (a wireframe) of how an app page will be laid out: where different content will be placed on the screen, how much space it will be given, and how it will connect to content on other app pages [35]. Wireframe testing allows the designer to elicit feedback about the structure and design of the app from people who might use it. Pandemic-imposed limitations caused us to hold the wireframe sessions via videoconference call. Eight participants were recruited via a convenience sampling approach; each person attended one of three videoconference calls. During the calls, participants were shown wireframes of each smartphone app screen. They were asked what they thought each screen was intended to convey, what functions they believed were available, and how the app would or would not help them reach health goals. Wireframes and follow up questions were adapted iteratively based on feedback from each session. We solicited feedback on items participants found confusing or problematic, with their recommendations for each of the targeted behaviors (diet, physical activity, and sedentary behavior). Wireframing was also performed for the attention control arm’s app, which addressed improving sleep and stress reduction.

##### Diet

Participants expressed confusion about the app’s home screen display for dietary impact. They were unclear about the food units referred to (serving size? points/credit?). To support food logging, participants recommended adding images to represent fruits and vegetables and to encourage variety. Other suggestions included providing recommendations for fruit and vegetable servings with each meal, adding app sound effects when logging fruit and vegetables, and showing a list of varying fruit and vegetables with points for each to help with setting and reaching goals.

##### Physical activity

Participants expressed lack of clarity about the units (minutes? calories?) presented on the app’s home screen display. They suggested adding an “other” option for those physical activities not represented in the app. Fitbits were recommended since they automatically track physical activity, including intensity. Participants thought it would be helpful if sedentary time could be automatically tallied by the app and if a prompt could be triggered to alert people when they had been sitting for hours at a time. Other general suggestions included adding more color to the app screen and updating the graphics to display more contemporary technology images.

##### Sedentary behavior

A fundamental question arose about how to define, measure, and display sedentary behavior. Participants came to a consensus that “sedentary behavior” meant sitting and/or sleeping for hours at a time. They also suggested that another term, such as “inactivity,” would be better understood in Appalachian Kentucky. The group also recommended that the app focus on setting a sedentary behavioral goal of fewer than 8 inactive hours a day. Consistent with participants’ confusion about the diet and physical activity units, they were uncertain whether the app’s display for sedentary time expressed the units in minutes or hours of sedentary behavior. These requests for greater clarity about the measurement units for the tracked behaviors were conveyed to our development team who modified the app’s design and function accordingly.

##### Sleep and stress

As the active control arm of the intervention, participants suggested that we clarify the conceptualization of stress and sleep. The consensus was that “quality sleep” means 6 to 8 h of uninterrupted sleep and that tracking should include logging the following: time to bed and time of awakening, when one falls asleep after going to bed, restroom trips through the night, and how rested one feels when waking in the morning. When asked what comes to mind when thinking about relaxation exercises for stress, CAB and community members named breathing exercises and meditation, along with activities such as stretching, walking, hiking, playing music, or taking a warm shower.

#### Usability survey

**Fig. 2:**
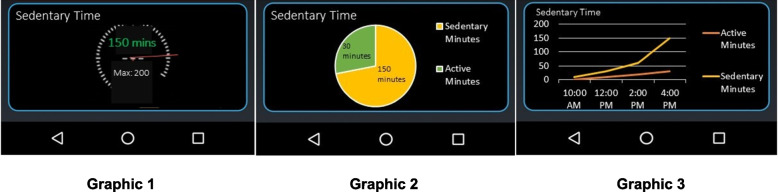
Graphical Representation Options for Sedentary Time. Legend. Graphic 1: Meter; Graphic 2: Pie chart; Graphic 3: Line graph

We again solicited input from our CAB members (*N* = 9) to refine remaining design questions, deploying a REDCap survey to verify design and syntax decisions (for example, should the targeted behavior be referred to as "sedentary" or "inactive"?). The survey was structured to solicit preferences for Fitbit use, background on prior health app use, interpretations of graphical representations of sedentary behavior, and preferences for graphics, shown in Fig. 2. The usability survey, with updated screenshots of the health app, was prepared in REDCap and shared via email with CAB members. Input suggested that (1) the pie chart (Graphic 2) was the preferred graphic, and (2) with the exception of sleep, all sedentary time should be demarcated as sedentary behavior.

#### Pilot testing

A brief pilot test was conducted among nine individuals to test the final smartphone app for functionality, to determine the feasibility of the recruitment strategies and protocol, and to gather final feedback to refine prior to the main trial. These participants, all of whom met the eligibility criteria for the upcoming main trial, were recruited using the adapted recruitment approaches (including social media, email/word-of-mouth, and flyers). The social media stand-alone study pages on Facebook, Twitter, and Instagram were launched simultaneously with an email campaign to the CAB and community contacts to request that they share and re-post information about the study. Throughout the pilot test, we posted social media messages five to seven times per week. These messages described study eligibility and activities, highlighting aspects of the study that FG participants had identified as appealing, including involving local coaches, technology, and financial incentives. Messaging also featured local community member involvement in the adaptation and project, introduced study personnel, provided information updates, and included a link to a study webpage where participants could volunteer by completing an online screener. We aimed to recruit participants for both the intervention condition (diet, physical activity, sedentary behavior) and the control condition (stress, sleep); thus, the messages were broad and referenced healthy lifestyles. During the pilot study, we “boosted” one message on Facebook, paying the online platform to increase the visibility of the message. All other messages relied on friends/followers to increase their visibility by interacting with the message.

Most interaction with social media occurred on the Facebook page, through which people interacted with our posts a total of 3,206 times. These interactions included reacting to a post, clicking on the survey link, and commenting. Neither Twitter nor Instagram generated many followers or interactions. Lack of engagement with these platforms is unsurprising, given high use of Facebook and low use of other social media platforms in rural areas [36].

A total of 218 people completed the eligibility survey during the pilot period and, of those, 165 (75.7%) indicated how they had learned about the study. Most (63.6%, *n* = 105) learned about the study from Facebook, although word-of-mouth (27.3%, *n* = 45) also contributed to recruitment. We launched our social media pages (Facebook, Instagram, Twitter) with messages that promoted the study and the study’s website. The social media messages included details of the study and directed those who were interested and potentially eligible to our website and screening survey.

##### Eligibility and screening

Consistent with the upcoming main trial, to be eligible for the pilot test, candidates had to meet the following criteria: being adults age 18 or older from any of the 54 counties in Appalachian Kentucky; willing to use their smartphone to record and modify diet, physical activity and sedentary behavior, and to wear a Fitbit for 12 weeks and intermittently thereafter for up to one year. Those with unstable medical conditions required physician approval to participate. To ensure the relevance of the treatment/behavioral change, we limited inclusion to those individuals who reported all of four problematic diet and physical activity behaviors: consuming < 4.5 cups of fruits/vegetables daily; engaging in < 150 min of moderate intensity physical activity weekly; and spending > 8 h daily of sedentary time (including sitting or lying down but not sleeping). We excluded individuals with cognitive impairment, active suicidal ideation, substance use disorder other than nicotine dependence, those at risk for adverse cardiovascular events with moderate intensity activity, and those with anorexia, bulimia, or other eating disorders or adhering to an incompatible dietary regimen. We also excluded those hospitalized for a psychiatric disorder within the past 5 years.

Three screening stages were used to assess eligibility. First, interested individuals used an online screener accessed through the study website. Those initially eligible were contacted by staff members via phone, text messaging, and/or email to schedule a telephone call to undertake the verbal informed consent process for screening and further determine eligibility. Third, individuals were emailed a link to the study’s baseline online questionnaire to be completed at home within one to two weeks. Once the baseline questionnaire was complete, those who remained eligible were permitted to select either an in-person or a remote baseline assessment.

##### Assessment and participant training

For the pilot test of the intervention, outcome data were collected in person at two time points: baseline and posttest. During baseline assessments, biometric data (blood pressure, lipids, A1C, waist circumference, BMI) were collected and additional surveys were completed. Fitbits, used in place of research-grade accelerometers (as advised by wireframe participants and the CAB due to Fitbit’s extra features and convenience), were distributed, and staff members trained participants on the health app, with specific focus on food logging and portion sizes. Training consisted of wearing a Fitbit and using the smartphone health app to record diet, physical activity, sleep, and stress data. The health coach and/or technician made a check-in/trouble-shooting call on the day after the participant began data entry. To ensure that participants were confident and competent in the use of the app, these data were viewed as practice tests rather than baseline data. The identical process was repeated one week later to collect the baseline data. All data were transmitted to the secure data platform via REDCap. Eligible participants were assigned to one of two intervention conditions: diet and activity or stress and sleep. A full description of the adapted intervention is described below.

##### Pilot results

Of the nine participants, six were assigned to the diet and activity condition and three were assigned to the stress and sleep condition. Eight of the participants completed all assigned coaching calls and one completed three of the five calls. Pilot participants noted several technical bugs in the smartphone application that were later addressed. Participants also requested improvements to the app such as the ability to see prior tracking episodes, push notifications to remind to track, and additional instruction and practice in logging food. Travel to the main site for the trial was noted as a concern for several participants which we have now addressed by providing a remote option. During exit interviews, pilot participants noted benefits of the interventions including enhancing insight regarding health behaviors, supporting accountability from the health coaches, and recognizing that healthy living is not striving for perfection but about attempting to make sustainable and long term healthy lifestyle decisions. Participants may not be involved in another diet, exercise, or weight control program. All other concomitant care and interventions are permitted during the trial.

### Description of the adapted MBC2 intervention

#### Overview

**Fig. 3:**
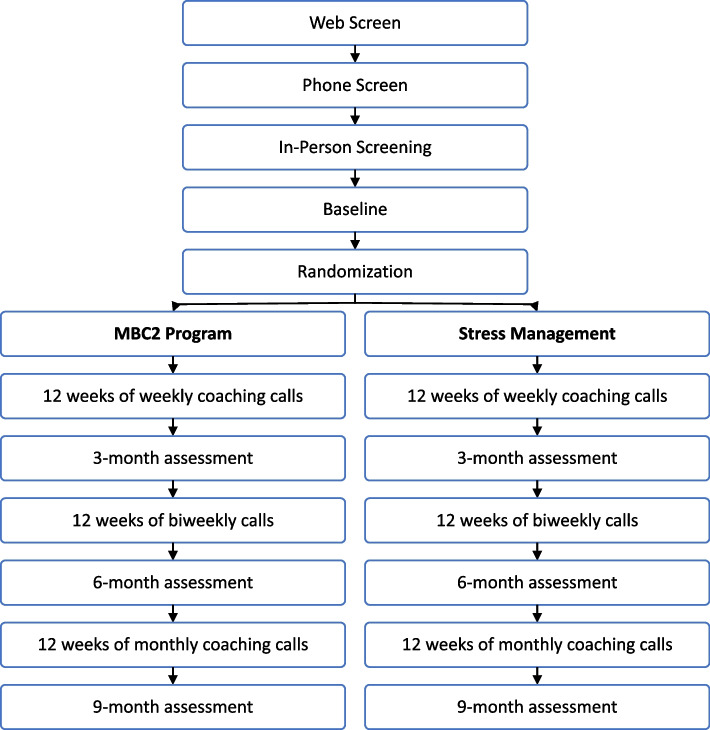
MBC2 for Rural Appalachians Flow Chart

After minor refinements to the app, we began implementing the MBC2 type 1 hybrid effectiveness-implementation trial. The main trial eligibility criteria, assessment content and schedule, and overall protocol will be identical to the pilot test protocol with several exceptions. In the main trial, participants will be randomized to one of two conditions; it lasts longer, data are collected at baseline plus three points in time, the intervention uses the revised app, and has been expanded to include iPhone as well as Android smartphone users. Figure 3 summarizes the protocol sequence, which includes the two-tiered assessment of eligibility, baseline assessment, randomization, 12 weeks of telephone-delivered coaching, and three outcome assessment periods in addition to a baseline (weeks 13, 26, and 39). In weeks 13–24, participants will enter the early maintenance phase with an in-person assessment and bi-weekly health coaching calls. During weeks 25–39, participants will enter the later maintenance phase by completing another in-person assessment and monthly health coaching calls. In week 40 (month 9), participants will complete the final assessment. Participants in both conditions (*N* = 350) will have the same number and duration of in-person assessments and telephone contacts over the 40 weeks. Only the coaching content and the visual displays will differ between the two conditions, reflecting the distinct behavioral foci.

#### Ethics and dissemination

All procedures were approved by the University of Kentucky’s Office of Research Integrity’s Medical Institutional Review Board (IRB). Protocol modifications have been and will be submitted to the IRB. In the future, we would submit any major modifications to scientific conduct to the study sponsor, the National Institutes of Health. Written informed consent was obtained from all participants prior to study procedures. Informed consent was obtained by the project manager and staff, all of whom will obtain needed certifications. All data are stored in a password-protected, firewalled system. While risks related to the intervention and attention control arms are anticipated to be minor, we maintain a data safety and monitoring plan and board (DSMB). All adverse events that do occur will be reported to PI who informs the IRB and DSMB. Participants may withdraw at their discretion. A publication plan and presentation plan has been developed and, as a community trial, results will be transmitted to relevant community partners during informational forums.

#### Adapted MBC2 intervention content

The program consists of four interconnected components: (1) app, (2) health coaching, including weekly lessons for participants, (3) Fitbit, and (4) behavioral incentives. These components encompass behavioral and implementation principles (effectiveness, scalability, and synergy), and they align with Goal Systems Theory. In addition to drawing upon Goal Systems Theory, the MBC2 intervention framework is enhanced by sociocultural and environmental considerations and blends well with the Consolidated Framework for Implementation Research [12, 29, 37].

##### App

This app incorporates a decision support system and display that helps participants monitor fruit & vegetable intake, moderate-vigorous physical activity (MVPA), and sedentary time relative to their daily target. The app also transmits this information to a web-based dashboard that is accessible to health coaches, enabling them to tailor telephone counseling to each participant’s progress and goals. During in-person training, participants are given materials to help them learn to estimate portion size. They also receive reminders that entries are time- and date-stamped to encourage prompt entry. The app automatically uploads data wirelessly, which enables detection of entry error or non-adherence to protocols. App features designed to support participants’ adherence to personal goals include (a) visual displays that provide feedback about intake and expenditure relative to targets throughout the day to guide self-regulation; (b) ability to access smartphone diet and physical activity databases to support decision-making about diet and physical activity choices; and, (c) use of stepped goals to facilitate incremental attainment of targets. In addition to app data, survey data about diet, physical activity, and sedentary behavior data will be collected at the assessment periods (baseline and weeks 13, 26, and 39) to assess intervention effectiveness.

##### Health coaching

Health coaching, evidence-based and strongly supported in our developmental research, comprises a central feature of the MBC2 intervention [33, 38]. For the main trial, coaches will (1) meet in person (or remotely, depending on the participant’s preference) with their assigned participant to help with training on the app and Fitbit and to set individualized goals; (2) provide weekly telephone counseling calls that address behavioral skill building and problem solving, tailoring recommendations to the resources of the community, participants’ preferences and stated challenges; (3) deliver online didactic lessons; (4) provide support and maintain accountability for self-monitoring behavior and initiating healthy change; and, (5) sustain support during the early maintenance phase (bi-weekly, weeks 13–24) and later maintenance phase (monthly, weeks 25–40).

Health coaches are recruited locally and must be comfortable with technology, knowledgeable about local context, and have excellent interpersonal and communication skills. In addition to our three current coaches, we anticipate recruiting and training three more coaches, allowing for the likely event that several coaches will leave or fail to meet fidelity criteria which will be assessed by anonymized reviewers. All coaches undergo extensive training in human subjects protection, app and Fitbit use, diet and physical activity, and brief, remotely-delivered health promotion techniques, including motivational interviewing (MI). All coaches are trained and certified through didactic sessions, role-play exercises, practice calls, and direct observation over several weeks until core competencies in MI and the research protocol are met. To maximize fidelity, all sessions are guided by scripts and checklists. Coaches avoid contamination across intervention conditions (e.g., coaches in the active MBC2 diet and activity treatment arm encourage physical activity but avoid addressing stress or sleep). Prior to intervening with study participants, all coaches must reach 100% proficiency by demonstrating core coaching skills and treatment fidelity benchmarks during three practice phone calls. Ongoing direct observation, immediate feedback after coaching sessions, and weekly clinical supervision via video conference will be implemented to prevent drift from approach and protocol over time. All subsequent coaching sessions will be audio-recorded, and a random 10% will be assessed quarterly for fidelity using a checklist. Because the primary focus of type 1 hybrid effectiveness-implementation studies is on effectiveness, fidelity will be maximized using these rigorous methods developed and used in prior MBC studies. In this context, intervention fidelity is required to ensure the quality of the delivered treatment (the trial’s independent variable), rather than treated as an implementation outcome.

##### Fitbit

Participants will be given a Fitbit Inspire 2 to wear daily on the non-dominant wrist during the intervention. To provide feedback to participants, the Fitbit data for MVPA, inactive minutes, and sleep will be sent via the application programming to be displayed on the study smartphone application as time spent in each behavior relative to goals.

##### Behavioral incentives

An incentive ($5) will be provided weekly to participants in both conditions if their behavioral average measuring standardized improvements for dietary and activity goals meets criterion for correct use of the app (for data collection) and attainment of behavioral targets (for diet and activity or for stress and sleep management). Consistent with our standard practice, participants will receive compensation for completing all major assessments (in-person and web-based at baseline and weeks 13, 26, and 39). The maximum total that participants will receive is approximately $200.

#### Active control condition: stress and sleep management

Since stress and sleep disturbances are so pervasive and impactful among rural Appalachian residents, this study arm is salient [39]. Protocols, including the use of the app and all assessments, will be identical between the two treatment arms except for intervention content. In the active control condition, goals pertain to stress and sleep management. As in the original MBC2 trial [12], participants in the control condition will focus on three behavioral goals: relaxation, stress reduction, and sleep. Control participants will be coached to perform three relaxation exercises per day (progressive muscle relaxation, mindfulness meditation, and self-hypnosis), and to achieve end goals of ≥ 7.5 h of sleep per day and a 30% reduction in stress over the 12-week intervention. Participants will use the 1 to 10 Subjective Units of Distress Scale (SUDS) to rate their stress [40]. During assessment periods (baseline, weeks 13, 26, and 39), both intervention and control groups will complete assessments for diet, physical activity, sedentary behavior, sleep, and stress.

Our biostatistician will randomize participants who demonstrate all four risk behaviors into either the diet and physical activity treatment arm (MBC2) or the sleep and stress arm (active control). Randomization will be achieved through a permuted block design using SAS v9.4 PROC PLAN. Sequences are not concealed. Allocation sequence and randomization are undertaken by the project biostatistician, Enrollment takes place by the project manager. Randomization will be stratified by sex, as we expect to recruit more women than men. We will employ the SPIRIT checklist (https://www.spirit-statement.org) to ensure standardization of all clinical trial elements.

### Outcomes

The primary effectiveness outcome for the main trial will be a continuous composite score that averages standardized improvements for each of the four targeted behavioral outcomes: two diet outcomes (increasing fruits and vegetables servings to ≥ 4.5 daily and decreasing saturated fat) and two activity outcomes (increasing physical activity to 150 min per week and reducing daily time spent on targeted recreational screen pastimes to 90 min per day) from baseline, week 13 (month 3) and week 39 (month 9). These outcome data will be obtained from the app data rather than from a separate food or activity frequency questionnaire. This primary outcome will be standardized on a common scale corresponding to an effect size by creating a Z- score. Secondary (exploratory) outcomes include change in biomarkers, including blood pressure, lipids, A1C, waist circumference, and BMI.

### Analyses

The trial was powered to have approximately 80% power to detect an intervention effect of 0.4 for the primary outcome when the sample size is 240 (120 per group), with a two-sided significance level of 0.05. To ensure adequate power after accounting for attrition and missing data, the primary analysis will include all 350 individuals randomized to treatment. We anticipate that (1) secondary comparisons will adjust for potential confounders, and (2) analyses for effects over time will be made using repeated measures. Including confounders reduces variability and can help to increase power. Further, because repeated measures are correlated, using the multiple measures in models also helps to increase power. For example, when the sample size is 120 participants per group (*N* = 240), a repeated measures analysis will have at least 80% power to detect the group*time interaction assuming that the groups start at baseline with no difference and then have effect sizes at weeks 13 (month 3) and 39 (month 9) of approximately 0.4 and 0.45, respectively.

## Discussion

Adapting, implementing, and evaluating existing evidence-based interventions for underserved populations has the potential to improve health equity with greater efficiency, effectiveness, and cost savings than initiating a program from scratch. However, few implementation studies employ a community-engaged process that incorporates cultural and contextual adaptation, thereby forfeiting the opportunity to develop appropriate, scientifically rigorous, sustainable interventions to achieve health equity [21]. Lack of cultural and contextual adaptation leads to inappropriate implementation, sacrificing the utility of programs where they are most needed [13, 21, 41].

In this article, we aimed to address the notable lack of explicit description on steps to adapt evidence-based interventions. Most advances in promoting the uptake and implementation of effective interventions have occurred in clinical or health systems settings [42, 43] and few provide extensive details on how this adaptation actually takes place. Given the iterative nature of adaptation, aligning with the context and culture of underserved populations requires an extensive and prolonged community engagement in the developmental phase of adaptation [22, 44].

We also have updated the literature to incorporate recent trends in technology use among rural residents, throwing off old assumptions of inadequate use, preference, or facility with such approaches. Ironically, increasing smart phone use, internet access, and broadband internet connectivity in rural areas contributes to sedentary behavior while also making the mHealth interventions that reach rural residents possible.

Our adaptation approach reveals the steps we took to ensure a balance between fit with the local population and adherence to standards of fidelity with the existing evidence-based intervention. While our iterative formative research and pilot testing has set the stage for culturally appropriate adaptation of this evidence-based intervention, it remains undetermined whether programs such as MBC2, developed and implemented in an urban environment, can remain efficacious when implemented in rural communities [45, 46]. Our next step is to implement and evaluate whether we have positively impacted participants’ health through this community-engaged, culturally, and contextually appropriate protocol.

## Supplementary Information

**Additional file 1. **SPIRIT 2013 Checklist: Recommended items to address in a clinical trial protocol and related documents*.

**Additional file 2. **World Health Organization Trial Registration Data Set: Make Better Choices 2 (MBC2) for Rural Appalachians.

## Data Availability

The datasets used and/or analyzed during the current study are available from the corresponding author on reasonable request. Information regarding data collection methods, descriptions of study instruments, and data collection forms is also available upon request.

## Abbreviations

Hemoglobin A1C level (A1C): A measurement of blood sugar levels, commonly used to diagnose and treat prediabetes and diabetes.
BMI: Body-mass index
CAB: Community Advisory Board
CFIR: Consolidated Framework for Implementation Research
COBRE: Center for Biomedical Research Excellence
FG: Focus groups
KII: Key informant interviews
KY: Kentucky
MBC2: Make Better Choices 2
mHealth: Mobile health
MVPA: Moderate vigorous physical activity
NIH: National Institutes of Health
REDCap: Research Electronic Data Capture
RUCA: Rural–urban commuting area
SUDS: Subjective Units of Distress Scale
