# Supplementary material for: Adaptation and study protocol of the evidence-based Make Better Choices (MBC2) multiple diet and activity change intervention for a rural Appalachian population
Source: BMC Public Health. 2022 Nov 8;22:2043. doi: 10.1186/s12889-022-14475-0 (PMC9643925; doi:10.1186/s12889-022-14475-0)
Supplement: Supplementary file 2 — Additional file 2. World Health Organization Trial Registration Data Set: Make Better Choices 2 (MBC2) for Rural Appalachians. [file 12889_2022_14475_MOESM2_ESM.docx]

**World Health Organization Trial Registration Data Set: Make Better Choices 2 (MBC2) for Rural Appalachians**

| **Data category** | **Information** |
| --- | --- |
| Primary registry and trial identifying number | ClinicalTrials.gov NCT04309461 |
| Date of registration in primary registry | 3 June, 2020 |
| Secondary identifying numbers | N/A |
| Source(s) of monetary or material support | National Institutes of Health/ National Heart, Lung, and Blood Institute |
| Primary sponsor | National Institutes of Health/ National Heart, Lung, and Blood Institute |
| Secondary sponsor(s) | N/A |
| Contact for public queries | Nancy Schoenberg, PhD  Department of Behavioral Science  University of Kentucky  Email: [nesch@uky.edu](mailto:nesch@uky.edu) |
| Contact for scientific queries | Nancy Schoenberg, PhD  Department of Behavioral Science  University of Kentucky  Email: [nesch@uky.edu](mailto:nesch@uky.edu) |
| Public title | Make Better Choices 2 (MBC2) for Rural Appalachians |
| Scientific title | Implementing the Make Better Choices 2 mHealth Energy Balance Intervention for Rural Appalachians |
| Countries of recruitment | United States |
| Health condition(s) or problem(s) studied | Health behavior |
| Intervention(s) | Active comparator:*Adapted Make Better Choices 2 (MBC2) diet and physical activity intervention* Attention control: stress and sleep management |
| Key inclusion and exclusion criteria | Ages eligible for study: ≥18 years Sexes eligible for study: both Accepts healthy volunteers: yes Inclusion criteria: adults residing in Appalachian Kentucky; willingness to use smart phone to record and modify diet and activity; willingness to wear an accelerometer (Fitbit); consume <4.5 cups fruit/vegetables daily; consume >8% daily calories from fat; engage in <150 minutes of moderate-intensity physical activity weekly; spend >90 minutes daily on non-work, non-education-related sedentary recreational use of screen time.  Exclusion criteria: unstable medical conditions; cognitive impairment; hospitalization for psychiatric disorder within the last 5 years; active suicidal ideation; substance use disorder other than nicotine dependence; at risk for adverse cardiovascular events with moderate-intensity activity; taking weight loss medication; trying to get pregnant, pregnant or lactating; active eating disorders; using mobility assistive devices; inability to read study materials |
| Study type | Interventional Allocation: randomized Intervention model: parallel assignment Masking: single (outcomes assessor) Primary purpose: PREVENTION  Phase: N/A |
| Date of first enrolment | 5 August, 2021 |
| Target sample size | 350 |
| Recruitment status | Recruiting |
| Primary outcome(s) | Change in diet and activity  (time frame: 3 months and 9 months)  A single composite MBC (Make Better Choices) score of four behaviors (fruit and vegetable intake, fat intake, physical activity, and sedentary time) will be used to assess change across multiple diet and activity behaviors. The MBC score, expressing each participant's overall change across the multiple diet and activity behaviors relative to baseline is calculated as the mean of four behavioral individual Z scores at each time point. Scores have an unlimited range; higher values represent greater healthy lifestyle improvement relative to the overall baseline distribution. |
| Key secondary outcomes | Change in biomarkers, including blood pressure, lipids, A1C, waist circumference, and BMI  (time frame: 3 months and 9 months) |
